# Conducting Research on the Internet: Medical Record Data Integration with Patient-Reported Outcomes

**DOI:** 10.2196/jmir.2202

**Published:** 2012-10-11

**Authors:** Elisa Cascade, Paige Marr, Matthew Winslow, Andrew Burgess, Mark Nixon

**Affiliations:** ^1^Digital Patient UnitQuintilesDurham, NCUnited States; ^2^Outcomes Health Information SolutionsAlpharetta, GAUnited States; ^3^QuintilesReadingUnited Kingdom

**Keywords:** Direct-to-patient study, patient-reported outcomes, observational research, medical record review, Internet recruitment, online patient communities

## Abstract

**Background:**

The growth in the number of patients seeking health information online has given rise to new direct-to-patient research methods, including direct patient recruitment and study conduct without use of physician sites. While such patient-centric designs offer time and cost efficiencies, the absence of physician-reported data is a key concern, with potential impact on both scientific rigor and operational feasibility.

**Objective:**

To (1) gain insight into the viability of collecting patient-reported outcomes and medical record information in a sample of gout patients through a direct-to-patient approach (ie, without the involvement of physician sites), and (2) evaluate the validity of patient-reported diagnoses collected during a patient-reported outcomes plus medical record (PRO+MR) direct-to-patient study.

**Methods:**

We invited a random sample of MediGuard.org members aged 18 to 80 years to participate via email based on a gout treatment or diagnosis in their online profiles. Interested members clicked on an email link to access study information, consent to participate electronically, and be screened for eligibility. The first 50 consenting participants completed an online survey and provided electronic and wet signatures on medical record release forms for us to obtain medical charts from their managing physicians.

**Results:**

A total of 108 of 1250 MediGuard.org members (8.64%) accessed study information before we closed the study at 50 completed surveys. Of these 108 members who took the screener, 50 (46.3%) completed the study, 19 (17.6%) did not pass the screening, 5 (4.6%) explicitly declined to participate due to the medical record requirement, and 34 (31.5%) closed the browser without completing the survey screener. Ultimately, we obtained 38 of 50 charts (76%): 28 collected using electronic signature and 10 collected based on wet signature on a paper form. Of the 38 charts, 37 cited a gout diagnosis (35 charts) or use of a gout medication (2 charts). Only 1 chart lacked any mention of gout.

**Conclusions:**

Patients can be recruited directly for observational study designs that include patient-reported outcomes and medical record data with over 75% data completeness. Although the validity of self-reported diagnosis is often a concern in Internet-based studies, in this PRO+MR study pilot, nearly all (37 of 38) charts confirmed patient-reported data.

## Introduction

The emergence of the e-patient—an individual seeking health information online—offers important opportunities to advance research. The ability to access targeted patient populations, coupled with technology to capture patient-reported outcomes data via the Internet, can reduce study timelines and cost, thus increasing the operational feasibility of real-world drug evaluation. These benefits are becoming increasingly important for observational research in light of growing demands for postapproval noninterventional studies, registries, and Risk Evaluation and Mitigation Strategy programs to evaluate real-world treatment effects.

In these direct-to-patient observational research designs, participant recruitment and data collection are conducted directly with patients rather than through clinic-based physician investigators. Although direct-to-patient studies initially focused on collection of patient-reported outcomes data only [1,2], these research designs have been evolving to incorporate clinical data from medical records as well as collection of genomic samples and laboratory data. In this paper, we report results from what we believe to be the first direct-to-patient research study involving collection of patient-reported outcomes data and clinical information extracted from patient medical records.

### Potential Limitations of Direct-to-Patient Studies

One of the greatest concerns associated with direct-to-patient observational studies is the quality of the information provided by patients outside of the investigator’s office. To investigate this issue, we conducted a pilot study in US gout patients using a design combining patient-reported outcomes and medical record data to evaluate the feasibility of recruiting a representative population of patients via the Internet, the willingness of physicians to provide medical record data, and the validity of self-reported diagnosis. We believe that this pilot study is the first to evaluate the ability to conduct observational direct-to-patient studies using a patient-reported outcomes plus medical record (PRO+MR) approach. As a result, we had no a priori hypothesis related to the proportion of charts that we could obtain or the validity of self-reported diagnosis in Internet research.

## Methods

### Study Design

This was a real-world, observational pilot study that combined patient-reported outcomes and medical records from gout patients recruited from an Internet database.

### Recruitment

We recruited participants from MediGuard.org, a free online service that monitors the safety of prescription medicines, over-the-counter medicines, and health care supplements for more than 2.5 million patients in the United States, United Kingdom, France, Germany, Spain, and Australia. MediGuard.org attracts members through online search engines and social media as well as outreach efforts with physicians, pharmacies, and health-related websites. Patients who enroll in the MediGuard.org service consent to be contacted about research opportunities as part of the registration process, and they double consent to participate in any individual study following receipt of an email invitation.

In July 2011, we invited US MediGuard.org members, aged 18 to 80 years, to participate in the study via email. Members whose MediGuard.org profiles cited a diagnosis of gout or use of a gout treatment (allopurinol or colchicine) were randomly selected for invitation to the study. Interested members clicked on an email link to access study information, provided their electronic signature for consent, and screened for eligibility based on a self-reported diagnosis of gout and willingness to release their medical records as part of the study design. Participants who completed the online survey and returned the paper medical record release form within 2 weeks were offered a US $50 MasterCard gift card as compensation for their time, to be mailed to the participant.

### Data Collection: Online Survey

The first 50 consenting participants were directed to complete an online survey of 20 questions that asked for information regarding disease and treatment experience, including time since first diagnosis of gout, specialty of treating physician, number of gout attacks in the previous year, number of physician visits for gout in the past year, satisfaction with treatment, and adherence to treatment regimens. Patients also reported demographic data such as age, gender, employment status, insurance status, household income, and highest level of education obtained. Prior to launching the online survey, we performed a quality control test to validate the functionality of the survey and data reporting tool.

### Data Collection: Medical Chart Authorization

After completing the online survey, participants also completed an electronic release form consenting to a medical record review. The purpose of accessing the patient’s medical record was to confirm the diagnosis of gout reported by the patient.

The medical chart release form asked participants to provide their physician’s name, address, specialty, and telephone and fax numbers, as well as the participant’s signature, name, telephone number, and date of birth. The participant’s personal information was electronically transferred to Outcomes Health Information Solutions (Alpharetta, GA, USA) in a secured manner to protect the participant’s personal information. After completing the electronic consent, participants were sent a paper copy of the medical record release form by regular mail. Participants were provided with a postage-paid, return-addressed envelope and requested to return the completed form within 1 week of receipt. Completed forms were mailed to Outcomes Health Information Solutions, a third-party contractor that specializes in health care abstraction and quality compliance programs.

### Medical Record Extraction

On receipt of the medical record release forms, Outcomes Health Information Solutions contacted patients’ managing physicians to schedule an appointment for chart extraction. Our approach to obtaining access to the chart was to first offer the physician the electronic medical record release. If the physician requested it, we subsequently provided a copy of the signed paper medical record release form when one was available.

According to the information provided in the medical record release form, Outcomes Health Information Solutions extracted data for the 2-year period from June 2009 to June 2011. Using jointly approved abstraction guidelines, nurses with clinical coding certifications reviewed the collected charts for evidence of gout. The abstraction guidelines defined acceptable physician notes and *International Classification of Diseases*, 9th revision (ICD-9) codes that could be used to support a diagnosis and excluded differential diagnoses as evidence of disease. If a diagnosis was not present in the 2-year chart data, the nurses examined the chart for mention of a prescription for colchicine or allopurinol, which are medications commonly used to treat gout. The date on which the paper medical record release form was received, the type of release form (paper or electronic) used for chart retrieval, the date when the chart was received, and the presence of a gout diagnosis or medication was entered into an Excel database by Outcomes Health Information Solutions, along with the de-identified patient study identification number.

### Validation of Patient-Reported Diagnosis

Diagnosis data obtained from the nurses were coded as confirmed, suspected, or missing based on the following formula: the patient’s self-reported diagnosis was considered confirmed if the medical chart contained a diagnosis; the patient’s self-reported diagnosis was considered suspected if the chart contained one of the medications commonly used for the treatment of gout; and charts containing neither a diagnosis nor medication were coded as missing.

### Statistical Analysis

Medical chart data elements were merged with information from the patient questionnaire based on the de-identified study identification number. We conducted all analyses on the aggregated data set.

Metrics related to the demographics and gout experience of the sample were analyzed. To evaluate the representativeness of the MediGuard.org population completing the study, we compared age and gender demographics with data obtained from Encuity Research’s Physician Drug and Diagnosis Audit (PDDA). According to Encuity’s website, “PDDA surveys more than 3,100 office-based physicians representing 29 specialties across the US. Physicians report on all patient activity during one typical workday each month. Once collected, the drug and diagnosis information is projected by region and specialty to estimate national activity for a universe of more than 460,000 physicians” [3]. We compared age and gender from our sample against Encuity’s PDDA data using Pearson chi-square tests. We were not able to access a data source to examine the representativeness of other gout-related study variables.

To describe the feasibility of chart extraction and diagnosis validation, we calculated two metrics related to the medical record process: the proportion of medical record release forms returned and charts received. In addition, we examined the type of release required to obtain the chart (electronic vs wet paper signature) and the status of the chart diagnosis (confirmed, suspected, or missing).

With respect to factors related to chart access, we compared the proportion of charts collected based on physician specialty, time since diagnosis, number of visits in the past year (3 items), and number of visits in the past year (binary). Binary variables (specialty and visits in the past year) were compared using the Fisher exact test. Time since diagnosis and the nonbinary physician visit variable were compared using the Cochran-Mantel-Haenszel statistic.

## Results

### Enrollment

MediGuard.org contains more than 8250 US members whose profile contains a diagnosis of gout or a medication likely to be used in the treatment of gout (eg, allopurinol or colchicine), or both. A total of 1250 emails were sent to MediGuard.org members, prompting 108 members (8.64%) to click on the email link to access more information about the study. Of the members screening for the study, 5 (4.6%) explicitly declined to participate due to the medical record requirement, and 34 (31.5%) closed the browser without completing the survey screener. If all individuals who closed the browser were to be included, the number of members who declined could be as high as 39 (36.1%).

### Sample Characteristics

A total of 50 members completed the study. It should be noted that the completion rate may have been higher, but the survey closed after the first 50 members had consented to participate. Table 1 displays demographic characteristics self-reported by the 50 participants completing the study. As seen in Table 1, participants represented a broad range of age, gender, employment status, insurance access, income, and education characteristics.

**Table 1:** Participants’ demographic characteristics (n = 50).

Characteristic		n	%	
**Age (years)**			
	<40	3	6%
	40–59	19	38%
	60+	28	56%
**Gender**			
	Male	34	68%
	Female	16	32%
**Employment status**			
	Employed	15	30%
	Not employed	35	70%
**Insurance status**			
	Insured—employer	17	34%
	Insured—public (Medicare, Medicaid)	27	54%
	Insured—Military/Department of Defense	3	6%
	Uninsured/self-pay	3	6%
**Household annual income (US $)**			
	<$25,000	14	28%
	$25,000–$34,999	7	14%
	$35,000–$49,999	7	14%
	$50,000–$74,999	9	18%
	$75,000–$99,999	1	2%
	$100,000+	9	18%
	Declined to answer	3	6%
**Highest level of education**			
	High school graduate	11	22%
	Some college	18	36%
	College graduate	13	26%
	Postgraduate studies	5	10%
	Declined to answer	3	6%

Comparison with demographic data from Encuity’s PDDA suggests that our sample had slightly more women than the general population of patients with gout (68% male in our sample vs 82% male in Encuity’s PDDA). We performed a Pearson chi-square test compare the gender in the sample against the Encuity’s PDDA data, and the difference was significant at the 5% level (*P *= .01). With respect to age, the sample was extremely similar to Encuity’s PDDA data: <40 years (6% in our sample vs 9% in Encuity’s PDDA), 40–59 years (38% sample vs 45% in Encuity’s PDDA), and 60+ years (56% sample vs 46% in Encuity’s PDDA). We performed a Pearson chi-square test comparing the sample against Encuity’s PDDA data with age pooled into two categories (<59, 60+), the difference was not significant at the 5% level (*P *= .17).

Table 2 summarizes self-reported characteristics of the participants’ gout condition. The majority of study participants (80%) were managed by a primary care physician. Two-thirds of patients in the gout sample had experienced a flare in the past year; however, only 50% of patients had visited their physician in the past year due to gout.

**Table 2:** Participants’ gout experience (n = 50).

Characteristic		n	%
**Time since first diagnosis of gout (years ago)**			
	<5	18	36%
	5–10	11	22%
	>10	21	42%
**Number of gout attacks in past year**			
	0	17	34%
	1	9	18%
	2	5	10%
	3	6	12%
	4+	9	18%
	I do not know/not sure	4	8%
**Number of physician visits for gout in past year**			
	0	25	50%
	1	10	20%
	2	4	8%
	3	2	4%
	4+	5	10%
	I do not know/not sure	4	8%
**Specialty of physician managing gout**			
	Primary care physician	40	80%
	Rheumatologist	7	14%
	Other	3	6%

### Medical Chart Data: Feasibility Evaluation

Table 3 displays metrics related to the medical record extraction process. As Table 3 shows, 42 of 50 participants (84%) who completed the online survey and electronic medical record release form also completed and returned the paper form. In total, we obtained 38 of 50 charts (76%): 28 (74%) were provided in response to the electronic medical record release; and 10 (26%) were provided in response to receipt of a wet signature on a paper form. The remaining 12 charts were not retrieved due to physician refusal (2 charts), the participant’s wet signature form was requested by the physician but not returned by the patient (2 charts), or the physician’s office did not complete the request by the time the study closed (8 charts).

In Table 4, we provide details on the number of charts accessed by characteristics such as physician specialty, time since diagnosis, and number of physician visits in the past year. While there were no significant differences based on these characteristics, a larger sample size might have yielded differences, particularly for the variable number of physician visits within the past year.

**Table 3:** Medical record process metrics.

Outcome		n	%
**Paper medical record release form returned with wet signature (n = 50)**			
	Yes	42	84%
	No	8	16%
**Chart received (n = 50)**			
	Yes	38	76%
	No	12	24%
**Type of release required (n = 38)**			
	Electronic	28	74%
	Paper	10	26%

**Table 4:** Factors related to number of charts access.

Type of access (n = 38)		n	%	*P *value
**By physician specialty^a^**				1.00
	Primary care (n = 40)	30	75%	
	Rheumatologist (n = 7)	6	86%	
**By years since diagnosis^b^**				.31
	<5 (n = 18)	13	72%	
	5–10 (n = 11)	7	64%	
	>10 (n = 21)	18	86%	
**By number of visits in past year** ^b^				.67
	0 (n = 25)	17	68%	
	1 (n = 10)	9	90%	
	2+ (n = 6)	4	67%	
**By number of visits in past year (binary)** ^a^				.48
	0 (n = 25)	17	68%	
	1+ (n = 16)	13	81%	

^a^ *P *values calculated by using Fisher exact test.

^b^ *P *values calculated using Cochran-Mantel-Haenszel statistic.

### Medical Chart Data: Diagnosis Validation

**Figure 1:**
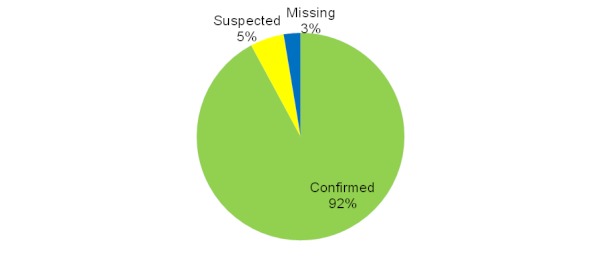
Medical record validation of self-reported diagnosis.

As Figure 1 shows, 35 of the 38 charts obtained (92%) contained a physician’s diagnosis of gout to confirm the patient’s self-reported diagnosis. An additional 2 charts (5%) contained notes regarding treatment with the gout medications allopurinol or colchicine; in these 2 cases, we considered gout to be likely and thus coded them as suspected. Only 1 chart (3%) was missing any diagnosis or notation of prescribed medication associated with gout.

## Discussion

Results of this pilot study are a positive step forward in confirming the viability of the PRO+MR direct-to-patient study approach and the validity of patient-reported outcomes when collected in this manner—a foundational step toward broader use of this design in collecting real-world, observational data. In this study, the findings demonstrated that patients could be recruited, screened, and enrolled directly from online patient communities for observational studies that collect patient-reported outcomes and medical record data, with more than 75% data completeness. Although there is concern that patients cannot be relied on to accurately report data in the absence of a physician investigator, in this PRO+MR pilot study, nearly all medical charts (37 of 38) confirmed the accuracy of a patient-reported gout diagnosis. Further, regarding the 1 chart that did not confirm diagnosis, it is unclear whether the respondent falsely claimed a diagnosis of gout or whether the chart was simply missing a note regarding patient diagnosis.

### Study Limitations

Although these findings have important implications for advancing direct-to-patient study design, the study has some limitations. First, we conducted our pilot PRO+MR study among a small sample of members with a single diagnosis (gout). Other studies are now being conducted in other conditions (eg, chronic obstructive pulmonary disease and rheumatoid arthritis) and with much larger sample sizes to determine whether the findings described in this paper are reproducible across patient types and physician practice specialties and settings.

Another limitation is that the medical record review in this study was restricted to a 2-year look-back in a single provider’s office for two primary fields: diagnosis and medications. Requesting more chart data or requiring a longer look-back period could have a negative impact on provider compliance. Additionally, while the current study extracted information from a single provider’s office, future study designs could require data that are dispersed across multiple care settings (eg, primary care and hospital records), also having a potential negative impact on data completeness. Developing a better understanding of the number of fields, type of information, and length of review period that is feasible in a medical record review will be a key objective of future projects.

It is also possible that unique characteristics of the MediGuard.org population influenced members’ interest and ability to participate in online clinical studies. For example, enrollment in MediGuard.org may self-select individuals with special interest in medical information that distinguishes them from the general population. Another distinguishing characteristic may be members’ facility in using the Internet. A higher level of Internet skills among members would also limit the generalization of findings to the broader population. However, according to a 2011 Pew report, 74% of all US adults use the Internet and 80% of these Internet users actively seek health information online, including information about a specific disease or treatment [4]. As the population ages and Internet-enabling technology continues to evolve with devices including smartphones and tablet devices, remaining concerns should diminish regarding the generalization of findings from direct-to-patient studies recruited from online patient communities.

Finally, there is the possibility that the methodology of combining patient-reported outcomes and medical record data resulted in a higher validation of self-reported diagnosis than would be achievable with patient-reported outcomes data alone. In other words, requiring a patient to consent to medical record extraction could have biased the sample toward patients who actually had the condition.

### Potential Benefits of Direct-to-Patient Studies

In addition to eliminating site-based costs, direct-to-patient studies offer major time and cost efficiencies. In traditional trials, estimates of recruitment costs range from US $10 to $1300 per completed participant [5,6], and recruitment delays can account for up to 45% of study delays [7]. The rise of online patient communities—such as PatientsLikeMe.com, CysticFibrosis.com, Inspire.com, and MediGuard.org—offers great recruitment advantages. For example, a recent study found that recruitment through MediGuard.org returned the highest percentage of candidates interested in a rheumatoid arthritis study at the lowest cost (US $4.82 per interested patient), compared with direct mail (US $86.28) and email generated by a third-party email list (US $195.65) [8].

Direct-to-patient studies can also overcome traditional recruitment barriers, such as patients’ lack of study awareness and limitations due to travel or site location. Patients learn about study opportunities through online communities and by searching social network sites; rather than traveling to an investigator’s office, patients participate in their own homes. Enhanced by greater awareness and wide geographical capture of study participants, and enabled by the high penetration of Internet access such as in the United States and Western Europe, direct-to-patient studies also offer a broader range of participant demographics and physician specialties and practice settings. This makes study results potentially more representative of the overall community of patients with a condition than do physician-centric studies, which are limited to the population of a few sites. Study retention is also likely to be higher in direct-to-patient studies because of the explicit alignment of patient incentives: the patient learns about the study directly, understands what is required to be compensated for participating in the study, self-consents to participate, and then self-reports study information.

Finally, direct-to-patient studies allow researchers in the United States to meet the Health Insurance Portability and Accountability Act requirements for maintaining patient information because the researchers obtain patients’ authorization to capture and store personal health information. Specifically, in direct-to-patient studies, researchers can engage patients directly via the Internet (no physicians involved); patients can choose to self-consent to participate in the study; patients voluntarily share identifiers to access their medical record; and patients provide an electronic signature and a wet signature on a paper form to have their medical record accessed. To maximize protection of personal health information, we use identifiers only to link survey and medical record data; once the linkage was completed, the study database was maintained in a de-identified format to minimize future risk of breach.

### Implications for Future Research

Confirmation of the validity of patient-reported data in observational, direct-to-patient remote studies is an essential step forward toward scaling up this method in real-world research. As direct-to-patient observational studies grow in number and size, experience and insights from these designs can be considered for integration into interventional clinical trials. While efforts to implement direct-to-patient approaches in interventional clinical trials, most notably the REMOTE study initiated by Pfizer in 2011 [9], are as yet inconclusive, knowledge gained through observational designs such as the PRO+MR model described here enables researchers to better identify study challenges and implement potential corrective solutions. Whether direct-to-patient studies are integrated in totality for interventional studies, or individual processes are adopted for recruitment, reporting, retention, and long-term extensions, one trend remains clear: the pool of e-patients will continue to grow globally, and harnessing the power of these patients offers the potential to drive a paradigm shift in clinical research.

## Abbreviations

ICD-9: International Classification of Diseases, 9th revision
PDDA: Physician Drug and Diagnosis Audit
PRO+MR: patient-reported outcomes plus medical record
